# Microbial carbon source utilization in rice rhizosphere soil with different tillage practice in a double cropping rice field

**DOI:** 10.1038/s41598-021-84425-0

**Published:** 2021-03-03

**Authors:** Haiming Tang, Xiaoping Xiao, Chao Li, Lihong Shi, Kaikai Cheng, Weiyan Li, Li Wen, Yilan Xu, Ke Wang

**Affiliations:** 1 Hunan Soil and Fertilizer Institute, Changsha, 410125 China; 2 Hunan Biological and Electromechanical Polytechnic, Changsha, 410127 China

**Keywords:** Microbiology, Environmental sciences

## Abstract

Carbon (C) plays an important role in maintaining soil fertility and increasing soil microbial community, but there is still limited information about how source utilization characteristics respond to soil fertility changes under double-cropping rice (*Oryza sativa* L.) system in southern China paddy field. Therefore, the effects of different short-term (5-years) tillage management on characteristics of C utilization in rice rhizosphere and non-rhizosphere soils under double-cropping rice field in southern China were investigated by using ^18^O incorporation into DNA. Therefore, a field experiment were included four tillage treatments: conventional tillage with crop residue incorporation (CT), rotary tillage with crop residue incorporation (RT), no-tillage with crop residue retention (NT), rotary tillage with crop residue removed as control (RTO). The results showed that soil microbial biomass C content with CT, RT, NT treatments were increased by 29.71–47.27% and 3.77–21.30% in rhizosphere and non-rhizosphere soils, compared with RTO treatment, respectively. Compared with RTO treatment, soil microbial basal respiration and microbial growth rate with CT treatment were increased 30.56%, 30.94% and 11.91%, 12.34% in rhizosphere and non-rhizosphere soils, respectively. The soil microbial C utilization efficiency were promoted with NT treatment. Compared with RTO treatment, the metabolic capacity of soil microorganism to exogenous C source with CT, RT and NT treatments were increased. The largest type of exogenous C source was saccharides, followed by amino acid and polymers, and complex compounds was the smallest. The redundancy analysis results indicated that tillage treatments significantly changed the utilization characteristics of soil microorganism to exogenous C source. Compared with RTO treatment, the grain yield of early rice and late rice with CT treatment were increased by 409.5 kg ha^−1^ and 387.0 kg ha^−1^, respectively. Therefore, the CT and RT treatments could significantly increase soil microbial biomass C content, but the NT treatment promote microbial C utilization efficiency in the double-cropping paddy field of southern China.

## Introduction

Soil microorganism plays a vital role in biogeochemical cycling, which are facilitated by soil disturbance and contributed to crop growth, soil nutrient cycling, and sustainability of soil productivity^1^. Soil organic carbon (SOC) content is affected by soil microorganism^2^. In the previous research, the results showed that SOC with no-tillage (NT) were lower than that of rotary and moldboard plow tillage with incorporated residue^3,4^. However, some studies showed that reduced tillage with rice residue retention increases the SOC content compared with conventional tillage (CT) and NT^5^. The soil microbial carbon (C) use efficiency (CUE) is usually defined as an important characteristics of soil microbial community metabolism^6,7^. Although the soil microbial community metabolism is importance for C cycling, there is still little information about the ecophysiology of microbial C cycling and the effects of different field management on soil microbial CUE^8^.

A higher CUE reflects a more efficient microbial growth and less C emission into the atmosphere through respiration^6,9^. There has closely relationship between soil microbial CUE and C nutrient, increases resource C/nutrient ratio generally reduce CUE because C excess relative to nutrient with respect to microbial biomass requirements, leads to respiration^10,11^. In agricultural system, soil microbial CUE is affected by different filed management, such as tillage, fertilizer regime, crop residue, irrigation pattern^7^. Soil tillage practice is a major factor in altering soil physical and chemical characteristics, nutrient cycling, which in turn modify soil microbial community composition and diversity^6,9^. Some studies found that microbial CUE was closely related to complex compounds and respiration rate per unit C assimilated^12^. Meanwhile, there was closely relationship between microbial CUE and organic C concentration and quality, microbial community composition^10^. Guo et al.^13^ results indicated that microbial metabolic activities were correlated to SOC within aggregates under conservation tillage condition. Thus, soil microbial CUE is affected by organic C quality, organic C components (polysaccharides, nitrogen rich compounds, soil humus compounds), C:N ratio, soil aggregation and its stability^5,7,14^. However, there is still not well understood the information about utilization characteristics of microbial C source and effects of different tillage with crop residue practice on soil microbial CUE in paddy field.

The available methods for investigated microbial CUE in paddy ecosystem have been accepted by more and more researchers^7^. In the previous research, these results showed that microbial CUE is estimated by microbial incorporation and respiration of labeled C from specific ^13^C-labeled substrate^15^. However, this method found it is very different CUE estimates for that this approach confounds microbial CUE with the specific substrate^7^. To overcome these problems, a novel method is suggested based on incorporation of ^18^O from water into DNA during growth, the increase in microbial biomass C in incubation time is calculated based on ^18^O-DNA^9^. Chen et al.^4^ results indicated that microbial CUE with organic input treatments were increased by 27–52% compared to chemical fertilizer treatment base on ^18^O-DNA method.

Rice (*Oryza sativa* L.) is the mainly crop in the world^16,17^. The early rice and late rice production system is the mainly crop system in southern of China^17^, and rotary tillage management is the main tillage practice in this region, because it has some advantages, such as creating reasonable soil structure, facilitating formation of soil aggregates, and high economic benefits. And the different short-term combined application of tillage (conventional tillage, rotary tillage and no-tillage) with crop residue practice has obvious influence on soil physicochemical characteristic (e.g., SOC content) and grain yield of rice^17^. We hypothesized that: (i) soil microbial CUE were affected with different tillage practice; (ii) metabolic capacity of soil microbe to exogenous C source were enhanced with crop residue practice, compared with without crop residue input practice. And a short-term field experiment with different tillage treatments under double-cropping rice system were set up in southern of China. Therefore, the objective of this study was (1) to explore rice rhizosphere and non-rhizosphere soil microbial CUE with different tillage treatments; (2) to investigate metabolic capacity of soil microbe to exogenous C source with various tillage treatments in the double-cropping rice system.

## Materials and methods

### Sites and cropping system

The experiment was begun in November 2015. It was located in Ningxiang County (28°07′ N, 112°18′ E) of Hunan Province, China. The climate condition (annual mean precipitation and evapotranspiration, monthly mean temperature) of this region, soil type and soil texture, the plough layer (0–20 cm) of physicochemical properties before this experiment, and crop system were described as by Tang et al.^17^.

### Experimental design

The experiment were included four tillage treatments: conventional tillage with crop residue incorporation (CT), rotary tillage with crop residue incorporation (RT), no-tillage with crop residue retention (NT), rotary tillage with all crop residue removed as control (RTO). The area of the each plots were 56.0 m^2^ (7 m × 8 m), and each treatments were laid out in a randomized complete block design with three replications. The detail information about the tillage practice, application of crop residue and chemical fertilizer, date of transplant and harvest of rice, and other field management were described as by Tang et al.^17^. The early and late rice seedling were manually transplanted to the paddy in April and July, and harvested with a combine in July and October, respectively. The cultivars of early rice were Xiangzaoxian 45 and the late rice were Xiangwanxian 13 during experimental stage, respectively.

### Soil sampling and sample preparation

Soil samples were collected in August 2019, at the maximum tiller stage of late rice. Rhizosphere soil was operationally defined as soil adhering to the total root after gentle shaking. The whole plant with their root were extracted from soil and after shaking off the loosely adhering soil, the rhizosphere soil were carefully collected. In order to obtain the enough rhizosphere soil for multiplicating, twenty plants were randomly selected from each plot, and these rhizosphere soil were pooled to form one composite sample^7^. Soil samples obtained 5–10 cm away from rice root were considered the non-rhizosphere soil. The non-rhizosphere soil adjacent to the rice plant were sampled at depth 0–20 cm. Correspondingly, one composite non-rhizosphere soil consisting of twenty cores was taken from each plot. Thus, three repeated samples of rhizosphere and non-rhizosphere soils with each tillage treatment were collected at sampling time. The fresh samples were placed immediately in ice box and transported to the laboratory. Plant root were removed by passing the sample through a 2-mm mesh sieve, and aliquots of the samples were then stored at room temperature until soil chemical properties analysis ((for pH, C/N, NH_4_^+^–N, NO_3_^–^N, soil organic carbon (SOC), and total nitrogen (TN)), at – 20 °C until molecular analysis. The samples were pre-incubated at 15 °C in aerated polyethylene bags for a total duration of six days before the beginning of the incubation, during which microbial carbon (C) utilization efficiency (CUE) and microbial biomass turnover was determined^7^.

### Soil laboratory analysis

#### Soil chemical properties analysis

Soil pH were investigated with a compound electrode (PE-10, Sartorious, Germany) by using a soil to water rate of 1: 2.5^18^. SOC and TN contents were measured by using an elemental analyzer (Carlo Erba 1110, CE Instruments) coupled to a Delta Plus isotope ratio mass spectrometer (Finnigan Mat) via a Conflo III (Thermo Fisher)^18^. Soil NH_4_^+^–N and NO_3_^–^N contents were investigated by extracting the soil with 0.01 M CaCl_2_ solution (1:10, w/v) for 30 min and then measured soil NH_4_^+^ and NO_3_^-^ contents by using a flow injection autoanalyzer (Fla Star 5000 Analyzer, Foss, Denmark)^18^. Soil clay was measured following the method as described by Zhao et al.^19^. Briefly, the soil clay sample were analysis of X-ray diffraction by using CuKa radiation and Philips Apd 3720 X-ray diffractometer equipped with focusing graphite monochromator. Soil microbial biomass carbon (MBC) and microbial biomass nitrogen (MBN) contents were investigated by using chloroform fumigation-extraction method^20^. Briefly, the pre-incubated soil were transferred to a clean desiccators and the residual CHCl_3_ evacuated. 80 mL of 0.5 M K_2_SO_4_ were added to the fumigated and non-fumigated soil sample, and 10 mL extract were taken to analysis SOC by using an automated C analyzer (Phoenix 8000) and 20 mL extract were digested and ammonium N were analysis calorimetrically by using a flow injection analyzer (Fiastar 5000).

#### Determination of soil CUE

After the pre-incubation period, soil microbial CUE were investigated based on incorporation of ^18^O from ^18^O-labeled water into microbial genomic DNA following the method were described as by Spohn et al.^9^. The respiration flux (C_Respiration_) were measured according to amount of carbon dioxide (CO_2_) carbon produce during incubation and duration of incubation period. The flux of C allocate to biomass production (C_Growth_) were measured by dividing amount of MBC produced during incubation and duration of incubation period. It were noted that production of MBC (growth) were not necessarily mean a net change in pool size (net growth)^9^.

Based on steady-state assumption, the amounts of C taken up by soil microbial biomass (C_Uptake_) were calculated as the following equation^9^:

1 $$ {\text{C}}_{{{\text{Uptake}}}} = {\text{C}}_{{{\text{Growth}}}} + {\text{C}}_{{{\text{Respiration}}}} $$

where C_Growth_ were the flux of C allocated to biomass production (growth), and C_Respiration_ were the flux of C allocated to the production of CO_2_ (respiration).

Microbial CUE were calculated as the following equation^9^:

2 $$ {\text{CUE}} = \frac{{{\text{C}}_{{{\text{Growth}}}} }}{{{\text{C}}_{{{\text{Growth}}}} + {\text{ C}}_{{{\text{Respiration}}}} }} $$

#### Characteristic of utilization of different type of exogenous carbon source

The C source was selected based on C source that were ecologically relevant to soil and that could be dissolved in water^21^. Microbial C metabolism were investigated by following the MicroResp method as described by Campbell et al.^21^ were used to measure CLPP. Briefly, the indicator dye with gel detector plate were including 20 ppm cresol red dye, 4 mM sodium bicarbonate and 240 mM potassium chloride set into 1% of the noble agar gel (150 μL per well). Soil sample were added to the 96-well microtiter deep well plate after 30 μL of each substrate had been dispensed. The metabolic capacity of soil microbe to different C source (CO_2_ production rate) within 4 h were calculated based on calibration curve of CO_2_ production rate and light absorption value of specific wavelength by indicator^21^.

CO_2_ production rate [μg (g h)^−1^] were calculated as the following equation^21^:

3 $$ {\text{CO}}_{{2}} \,\, {\text{production}} \,\, {\text{rate}} = \frac{{{\text{CO}}_{{2}} \times {1}0000 \times L \times {12} \times {273}}}{{{22}.{4} \times \left( {{273} + T} \right) \, \times M \times t}} $$

where *T* were the incubation temperature (25.0 °C), *L* were the volume of per hole deep orifice plate (945 μL), M were dry weight of soil (g), and *t* were incubation time (4 h).

#### Rice grain yield

Rice grain yield in each plot were measured at mature stages of early and late rice in 2019, and 1 m^2^ area of each plot were collected to calculate the dry weight of rice grain yield.

### Statistical analysis

All the results were reported as the means and standard errors (SE). All statistical analysis were calculated by using the SAS 9.3 software statistical package^22^. The relationship between soil chemical properties and soil microbial C source utilization rate were calculated by using redundancy analysis (RDA). The correlation between different variables were analysis by using the type II scaling (correlation plots) of RDA. The RDA were performed by using Canoco software statistical package (Version 3.20). The investigated data of each treatment means were compared by using one-way analysis of variance (Anova) following standard procedures at the 5% probability level.

## Results

### Grain yield of rice

**Figure 1:**
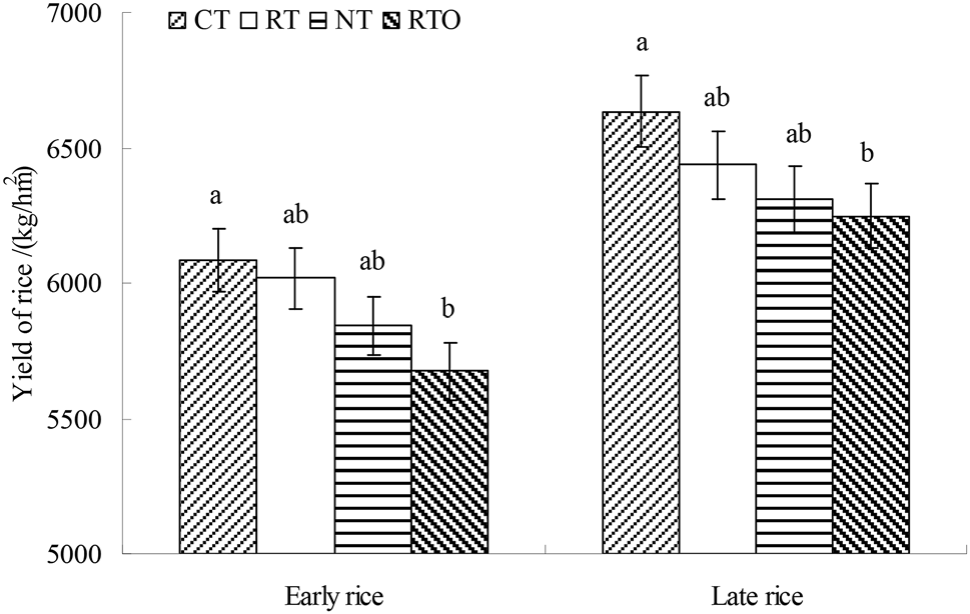
Grain yield of rice with different tillage treatments. *CT* conventional tillage with crop residue incorporation, *RT* rotary tillage with crop residue incorporation, *NT* no-tillage with crop residue retention, *RTO* rotary tillage with crop residue removed as control. Error bars were represented standard error of mean. Different smaller letters were indicated significantly difference at *P* < 0.05 level.

The grain yield of rice after 5-years of cropping under different tillage treatments were shown in Fig. 1. The grain yield of early rice with CT treatment were higher (*p* < 0.05, p = 0.045) than that of RTO treatment. Compared with RTO treatment, the grain yield of early rice with CT treatment increased by 409.5 kg ha^−1^. Meanwhile, the results showed that grain yield of late rice with CT treatment were higher (*p* < 0.05, p = 0.041) than that of RTO treatment. Compared with RTO treatment, the grain yield of late rice with CT treatment increased by 387.0 kg ha^−1^, respectively. The grain yield of early and late rice with RT and NT treatments were higher than that of RTO treatment, but there was no significantly (*p* > 0.05, p = 0.065) difference in grain yield of early and late rice between RT, NT and RTO treatments (Fig. 1).

### Soil microbial carbon utilization efficiency

**Figure 2:**
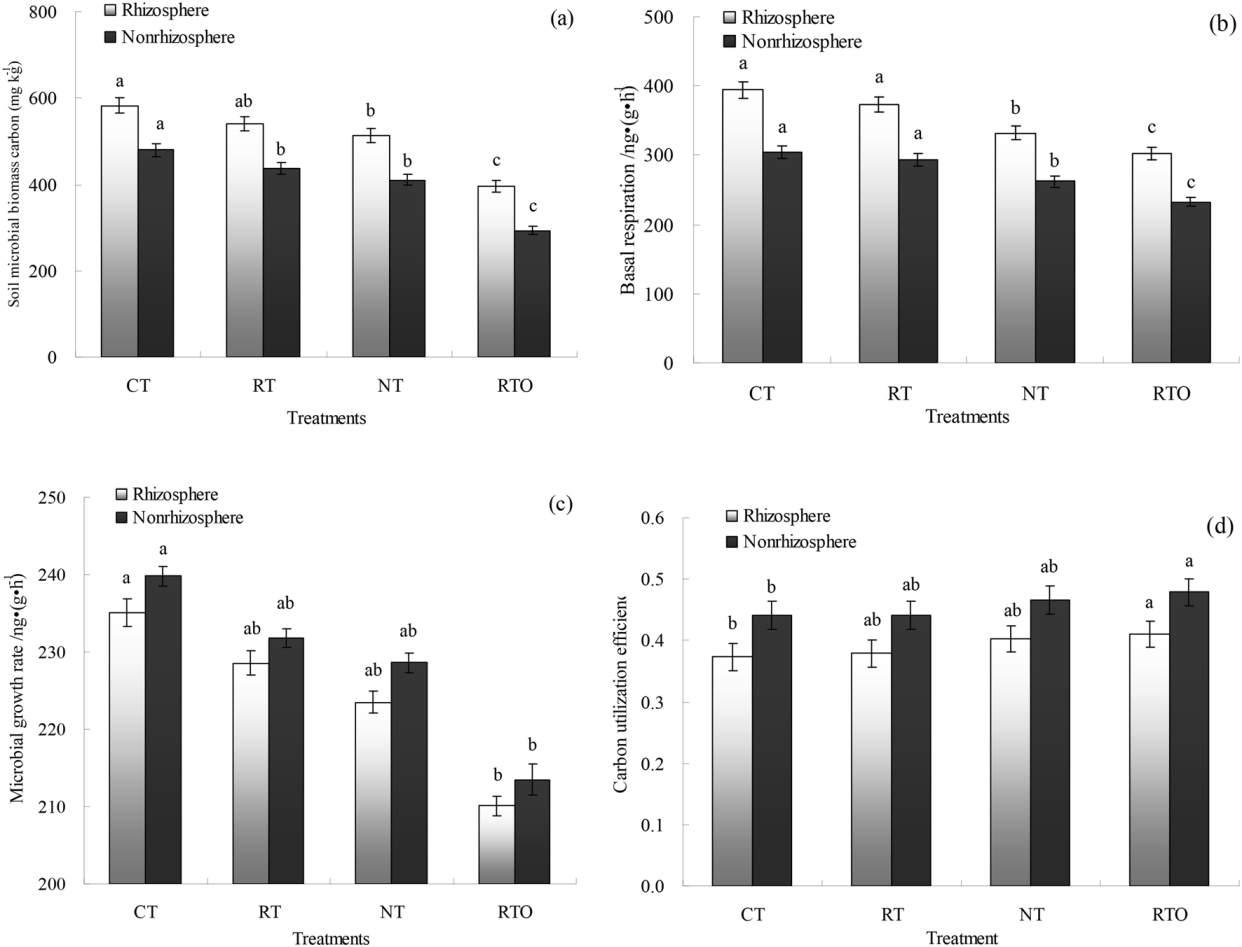
Soil microbial distribution in rhizosphere and non-rhizosphere soils with different tillage treatments. (**a**) Soil microbial biomass carbon. (**b**) Basal respiration of soil microorganism. (**c**) Soil microbial growth rate. (**d**) Soil microbial carbon utilization efficiency. *CT* conventional tillage with crop residue incorporation, *RT* rotary tillage with crop residue incorporation, *NT* no-tillage with crop residue retention, *RTO* rotary tillage with crop residue removed as control. Error bars were represented standard error of mean. Different smaller letters were indicated significantly difference at *P* < 0.05 level.

In rhizosphere and non-rhizosphere soils, the soil microbial biomass carbon (MBC) content with CT, RT, NT and RTO treatments was from 293.13 to 582.74 mg kg^−1^. The rhizosphere soil MBC content with CT, RT, NT and RTO treatments were significantly higher (*p* < 0.05, p = 0.037) than that non-rhizosphere soil. And the rhizosphere and non-rhizosphere soils MBC contents with CT, RT and NT treatments were significantly higher (*p* < 0.05, p = 0.039) than that of RTO treatment (Fig. 2a).

In rhizosphere and non-rhizosphere soils, the soil microbes basal respiration with CT, RT, NT and RTO treatments was from 232.45 to 394.45 ng (g h)^−1^. The rhizosphere soil microbe basal respiration with CT, RT, NT and RTO treatments were significantly higher (*p* < 0.05, p = 0.039) than that of non-rhizosphere soil. And the rhizosphere and non-rhizosphere soils microbe basal respiration with CT and RT treatments were significantly higher (*p* < 0.05, p = 0.035) than that of NT and RTO treatments, but there were no significantly difference (*p* > 0.05, p = 0.062) in soil microbe basal respiration between RT and CT treatments (Fig. 2b).

The results showed that range of rhizosphere and non-rhizosphere soils microbial growth rate (C_Growth_) with CT, RT, NT and RTO treatments was 210.12 to 239.83 ng (g h)^−1^. And the non-rhizosphere soil C_Growth_ with different tillage treatments were significantly higher (*p* < 0.05, p = 0.041) than that of rhizosphere soil. In rhizosphere and non-rhizosphere soils, the soil C_Growth_ with CT treatment were higher (*p* < 0.05, p = 0.035) than that of RTO treatment, but there was no significantly (*p* > 0.05, p = 0.061) difference in soil C_Growth_ between CT and RT, NT treatments (Fig. 2c).

In rhizosphere and non-rhizosphere soils, the CUE of soil microbial with different tillage treatments was 0.37 to 0.48 ng (g h)^−1^. The CUE of soil microbial in non-rhizosphere soil with different tillage treatments were significantly higher (*p* < 0.05, p = 0.040) than that of rhizosphere soil. In rhizosphere and non-rhizosphere soils, the CUE of soil microbial with RTO treatment were higher (*p* < 0.05, p = 0.042) than that of CT treatment, and the CUE of soil microbial were increased with NT treatment, but there was no significantly (*p* > 0.05, p = 0.064) difference in CUE of soil microbial between NT and CT, RT treatments (Fig. 2d).

### Characteristics of utilization of different type of exogenous carbon sources

**Figure 3:**
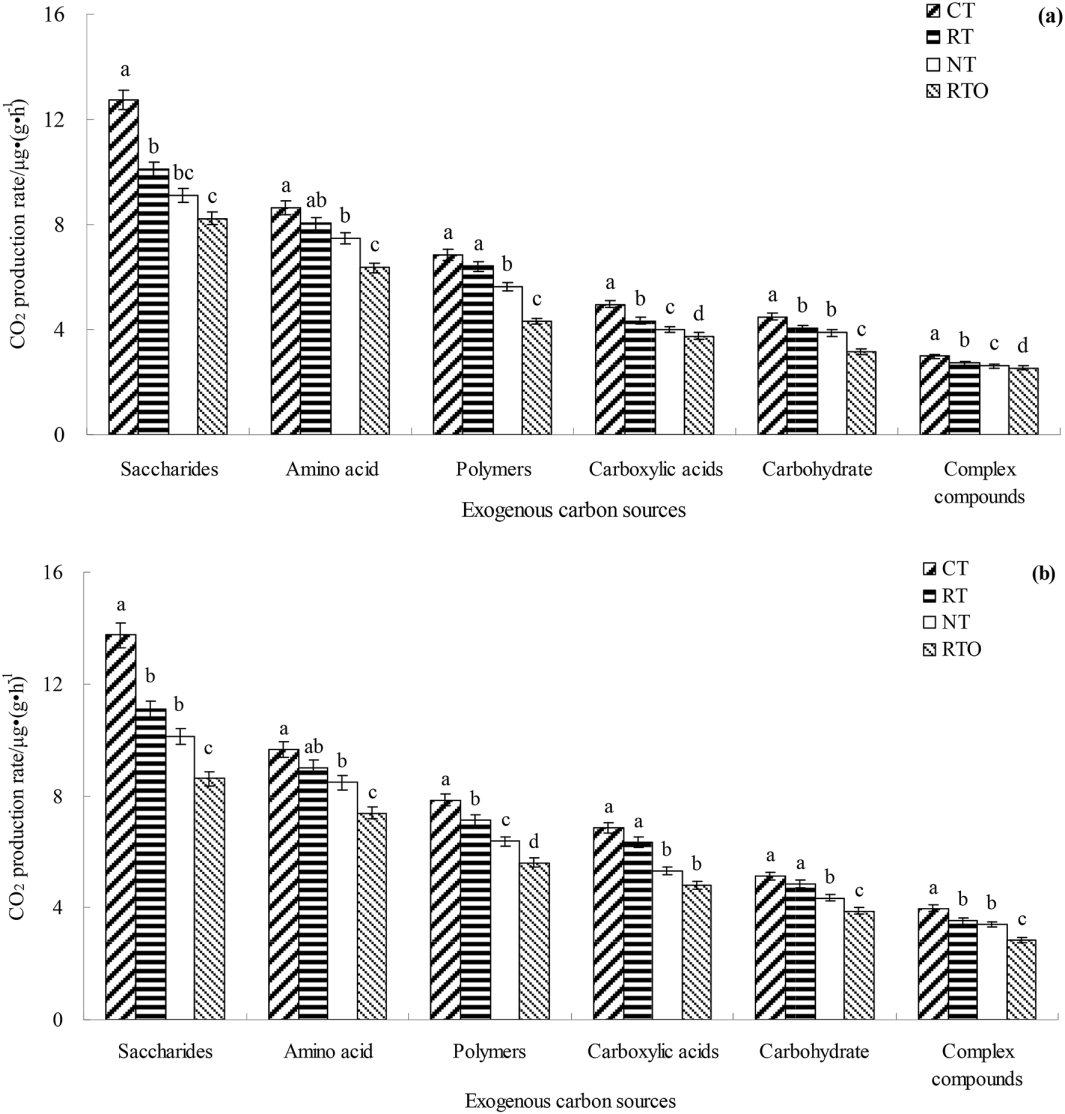
Characteristics of utilization of different type of exogenous carbon source by rhizosphere and non-rhizosphere soils microorganism with different tillage treatments. (**a**) Rhizosphere soils. (**b**) Non-rhizosphere soils. *CT* conventional tillage with crop residue incorporation, *RT* rotary tillage with crop residue incorporation, *NT* no-tillage with crop residue retention, *RTO* rotary tillage with crop residue removed as control. Complex compounds were including thiamine, imidazole, niacin, cinnamon, coumarin, catechin and quercetin.

The metabolic capacity of soil microorganism to exogenous C source in non-rhizosphere soil with different tillage treatments were higher (*p* < 0.05, p = 0.041) than that of rhizosphere soil (Fig. 3). Compared with RTO treatment, the metabolic capacity of soil microorganism to exogenous C source in rhizosphere soil were increased with CT, RT and NT treatments. In different type of exogenous C source, the average utilization rate of saccharides with different tillage treatments were higher than that of complex compounds (thiamine, imidazole, niacin, cinnamon, coumarin, catechin and quercetin). In rhizosphere and non-rhizosphere soils, different type of exogenous C source with CT, RT, NT and RTO treatments were utilized following decreasing order: saccharides > amino acid > polymers > carboxylic acids > carbohydrate > complex compounds.

**Figure 4:**
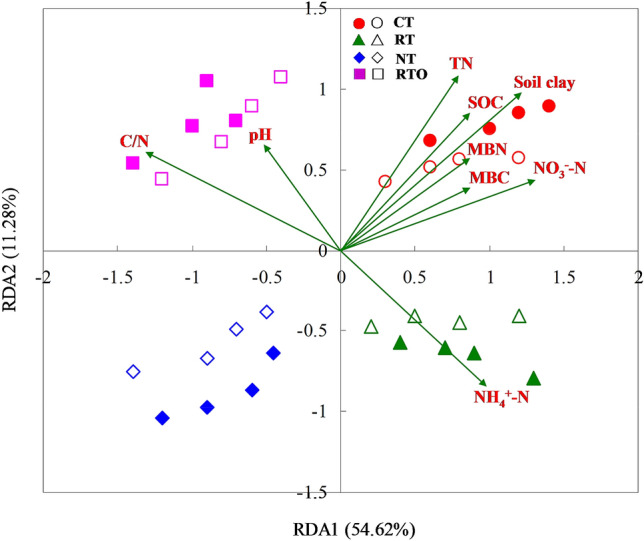
Redundancy analysis of soil microbial carbon source utilization rate and soil chemical properties. *CT* conventional tillage with crop residue incorporation, *RT* rotary tillage with crop residue incorporation, *NT* no-tillage with crop residue retention, *RTO* rotary tillage with crop residue removed as control. Solid icon and hollow icon were indicated rhizosphere and non-rhizosphere soils, respectively. *SOC* soil organic carbon, *TN* soil total nitrogen, *MBC* soil microbial biomass carbon, *MBN* soil microbial biomass nitrogen.

Considering the whole microbial C source utilization rate, the results revealed there was a significantly correlation between microbial C source utilization rate and soil chemical properties (Fig. 4). In addition, the soil chemical properties can explain the variation (54.62%) in microbial C source utilization rate between CT, RT, NT and RTO treatments. Under different tillage treatments, NT treatment were separated from RT treatment, CT treatment were separated from RTO treatment. The results indicated that NT and RTO treatments were separated from CT and RT treatments, indicated that utilization characteristics of soil microorganism to exogenous C source were significantly changed with different tillage treatments. The soil chemical properties was significantly correlated with utilization characteristics of soil microorganism to exogenous C source including the soil clay, soil C/N, TN, SOC, NH_4_^+^–N, and NO_3_^–^N contents.

## Discussion

In the present study, the results indicated that rhizosphere and non-rhizosphere soils MBC content were increased with combined application of tillage with crop residue practice, compared without crop residue input practice. The reason maybe that soil available nutrient were increased by application of crop residue, which provided C substrate and nutrient for the growth and reproduction of soil microorganism, and then increased soil microbial growth rate^13^, MBC were increased by 29.71–47.27% and 3.77–21.30% in rhizosphere and non-rhizosphere soils, compared to without crop residue input practice. Meanwhile, the soil MBC content with CT treatment were higher than that of the other treatments, the reason may be that crop residue were incorporated into soil plough layer with CT practice, providing a large amount of available substrate for soil microbial growth, and the soil microbial activity were increased. On the other hand, rice root exudation and root activity were also promoted under conventional tillage condition for that it were provided favorable soil environment for rice growth^23^. This result indicated that soil MBC content with RT treatment were also higher than that of NT treatment, the reason may be that crop residue were incorporated into 0–10 cm soil layer when tillage at depth of 8–10 cm, the crop residue providing available substrate for soil microbial growth, the root of rice and rhizosphere soil microbial activity were enhanced^24^. And the basal respiration of soil microorganism were increased under application of crop residue condition, the reason maybe that decomposable crop residue was the main C source for microbial utilization, and there were significantly differences in the content of decomposable organic C with different tillage treatments^13^. On the other hand, nature of rice root exudates may be inducing a positive stimulating effect of SOC decomposition, which in turn increases soil microbial respiration^25^. In this study, the C_Growth_ with combined application of tillage with crop residue treatments were higher (*p* < 0.05) than of without crop residue input treatment, the reason maybe that there were significantly differences in SOC content among different tillage treatments, which may lead to significantly differences in available C source of soil microorganism^17^, which was similar with Wang et al.^4^.

In this study, the results showed that range of rhizosphere and non-rhizosphere soils microbial CUE with different tillage treatments, which was in agreement with Manzoni et al.^6^. And the CUE of rhizosphere soil microbial were lower than that of non-rhizosphere soil, the reason maybe that metabolic capacity of microorganism were higher in non-rhizosphere soil, thus the distribution of respiration C by soil microorganism were higher than that of growth C^7^. On the other hand, rhizosphere soil C content were increased in the process of root system absorbs nutrient, and microbial CUE were decreased with the increase of C content^9^, which was similar with previous research^7^. There was no significantly difference in CUE of non-rhizosphere soil microbial with CT, RT and NT treatments, the reason may be that physiological environment of non-rhizosphere soil was relatively stable, which was consistent with previous research^26^. In this study, the higher CUE of soil microbial with RTO treatment than that of CT, RT and NT treatments consistent with our Hypothesis 1 that soil microbial CUE were changed under combined application of tillage with crop residue condition (Fig. 2), the main reason was that soil C nutrient ratio and carbon dioxide (CO_2_) through overflow respiration were increased, soil C nutrient ratio was benefit to meet the nutritional needs of soil microorganism^10^, and thus lower soil microbial CUE under application of crop residue condition^9^. In the present study, the yield of rice reflects changes in soil microbial CUE and MBC content (Fig. 1). The yield of early rice and late rice with CT treatment were higher than that of RTO treatment. The soil microbial CUE and MBC content with CT treatment were higher than that of RTO treatment. Similar results reported that rice yield and soil physicochemical properties of paddy field were increased through application of crop residue and tillage practice^3,4^.

The bioavailability and function diversity of soil microbe can express by C metabolism of soil microbial community^27^. And the ability of soil microbial community to utilize C source is usually reflected by average CO_2_ production rate^7^. In this study, the results showed that metabolic capacity of soil microbe to exogenous C source with combined application of tillage with crop residue treatments were higher than of without crop residue input treatment. The reason maybe that crop residue contains a large number of microorganism and organic C source, and thus the metabolism of microorganism to C source were promoted when crop residue were incorporated into plough layer with tillage treatment. Therefore, this results was consistent with our Hypothesis 2 that metabolic capacity of soil microorganism to exogenous C source would be higher with tillage and crop residue management than that of without crop residue input management. And the metabolic capacity of soil microorganism to exogenous C source with CT and RT treatments were higher than NT treatment, indicated that soil C/N were increased, soil microbial activity and decomposition rate were decreased, and thus soil microbial metabolic ability to C source were decreased under no-tillage condition^28^. In this study, the results showed that saccharides, amino acids and polymers were the main C source used in crop residue treatments of soil microbiological utilization, which was similar with the previous study^29^. Meanwhile, our results indicated that average utilization rate of carboxylic acids were higher than that of complex compounds, suggested that carboxylic acids C source is an vital energy source for soil microbe growth and metabolism^30^. On the other hand, the reduce of complex compounds under interaction of different extra-cellular enzyme^7,31^, more C and energy source were needed during soil microbe growth and multiplying, therefore, the utilization rate of complex compound were decreased^31,32^. In the present study, the correlation between soil microbial C source utilization rate and soil physicochemical characteristic were analysis by using type II scaling of RDA. The RDA results showed that there was obvious difference in metabolism of soil microorganism to exogenous C source between tillage treatments, indicated that tillage treatments has greatly changed the soil environment, crop residue and microorganism were introduced, and then significantly changed the utilization characteristics of soil microorganisms to exogenous C source.

In the present study, the soil microbial growth rate were increased, but the soil microbial CUE were decreased under combined application of tillage with crop residue condition. Furthermore, metabolic capacity of non-rhizosphere soil microorganism to exogenous C source were higher than that of rhizosphere soil (Fig. 3), which was inconsistent with the results of soil basic respiration based on ^18^O–H_2_O method. The reason may be that MicroResp method mainly monitors microbial decomposition of exogenous C, which was not distinguish the excitation effect, but ^18^O–H_2_O method mainly monitors microbial decomposition of soil C. On the other hand, rhizosphere soil were adaptability to exogenous C source input (root exudates) condition. Meanwhile, the positive stimulation effect of decomposition of crop residue C in non-rhizosphere soil were induced with input of exogenous C source, and thus microbial mineralization of organic C were enhanced^25,33^.

In conclusion, this study indicated that characteristics of C source utilization in rhizosphere and non-rhizosphere soils were significantly affected by short-term tillage management in a double-cropping rice field. Our results showed that combined application of tillage with crop residue practices were promotes soil microbial biomass C content and microbial growth rate in both rhizosphere and non-rhizosphere soils, whereas application of no-tillage with crop residue retention practice were promotes soil microbial C utilization efficiency. The metabolic capacity of non-rhizosphere soil microorganism to exogenous C source were higher than that of rhizosphere soil under different tillage condition. And the utilization rate of saccharides, amino acid, polymers, carboxylic acids and carbohydrate by soil microorganism were increased with combined application of tillage with crop residue practice. There is an obvious difference in characteristic of C source metabolism between application of tillage treatments and no-tillage treatment. Moreover, higher grain yield of rice was directly attributed to improvement in soil microbial biomass C content and soil microbial C utilization efficiency. However, future studies were needed to investigate how changes of C source utilization soil microbial structure under different tillage practice influence on ecological function of rhizosphere microorganism.

## Conclusions

In the double-cropping rice field, this result showed that characteristic of carbon source utilization in rhizosphere and non-rhizosphere soils were obvious changed by taken short-term tillage practice. And the soil microbial biomass carbon content and microbial growth rate in rhizosphere and non-rhizosphere soils were increased under combined application of tillage with crop residue condition. Soil microbial carbon utilization efficiency were increased under no-tillage with crop residue condition, whereas soil microbial carbon utilization efficiency were decreased under combined application of tillage with crop residue condition. The non-rhizosphere soil microbe metabolic capacity of exogenous carbon source were higher than that of rhizosphere soil. And the combined application of tillage with crop residue management promote soil microbe utilization rate of saccharides, amino acid, polymers, carboxylic acid and carbohydrate. There were significantly differences in carbon source utilization characteristic of soil microbial community between conventional tillage, rotary tillage and no-tillage treatments. However, future studies still need to investigate how change of carbon source utilization soil microbial structure influence on ecological function of rhizosphere soil microorganism under long-term tillage condition.
